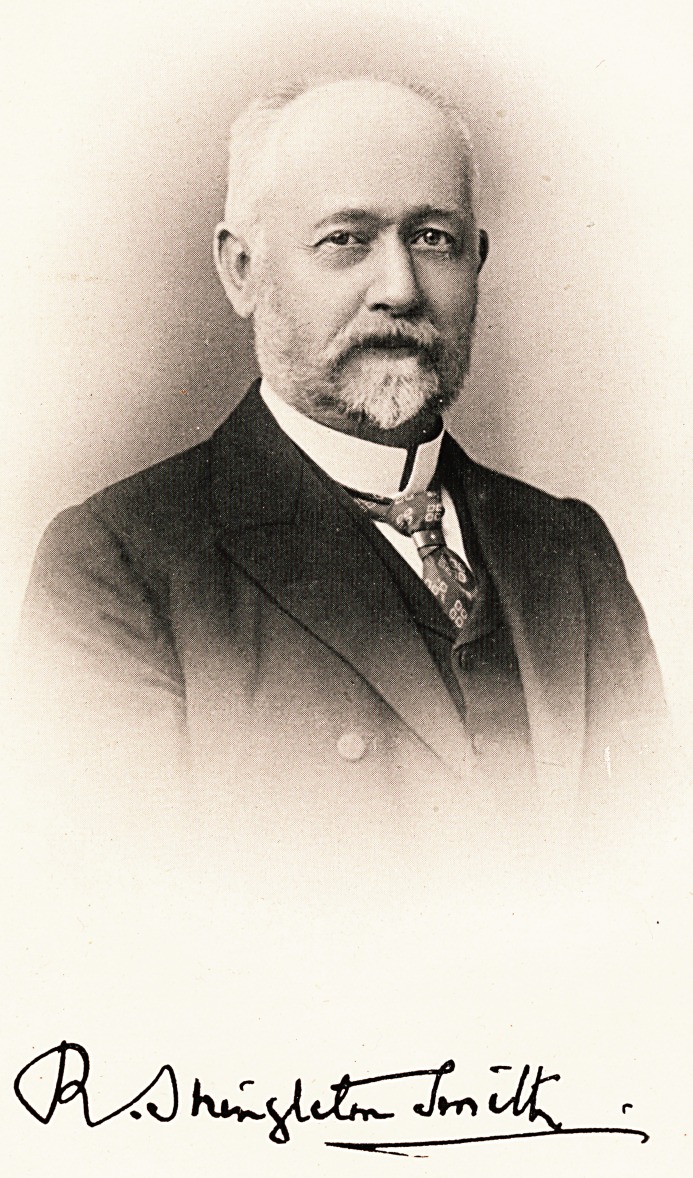# Robert Shingleton Smith, M.D., B.Sc., F.R.C.P.

**Published:** 1913-03

**Authors:** 



R. Shingleton Smith

### The Bristol Medico-Chirurgical Journal.

*"Scire est nescire, nisi id me*
*Scire alius sciret."*

MARCH, 1913.

### ROBERT SHINGLETON SMITH, M.D., B.Sc., F.R.C.P.

EDITOR, 1892–1912.

---

THE resignation of the Editor of this *Journal* after twenty years' service is an epoch in its career impelling us to recall the generous devotion, the energy and skill with which it has been piloted throughout this long period, and to look forward in the hope that its interest and usefulness may not suffer in the future.

In accepting this responsible office, the new Editor is grateful for the continued co-operation of his colleagues on the Editorial Committee with whom he has worked for many years as Assistant Editor, thus mitigating his responsibility, while enhancing the honour conferred on him.

The excellent portrait of Dr. Shingleton Smith will afford pleasure to his many friends.

On Saturday evening, January 18th, a very pleasant gathering of medical men took place at Fortt's Restaurant, at which Dr. Shingleton Smith was entertained at dinner by his old colleagues and friends, who took the opportunity of his resignation of the Editorship of the *Bristol Medico-Chirurgical Journal* to show their esteem and regard for him, and to make him a presentation in recognition of his services to scientific medicine in Bristol.

Professor Walter C. Swayne, the President of the Bristol Medico-Chirurgical Society, took the chair at the dinner, at which the attendance was thoroughly representative of the local profession, and included nine past Presidents of the Society. After the usual loyal toasts, Professor Swayne called upon Mr. Nelson C. Dobson to propose Dr. Shingleton Smith's health.

Mr. Dobson made a most eloquent and effective speech, as he can always be trusted to do on such occasions, and admirably expressed the respect and regard felt for Dr. Shingleton Smith by his medical brethren. He referred to the founding of the Bristol Medico-Chirurgical Society, which issued on the initiative of Dr. Shingleton Smith, aided by himself and Dr. Spencer, and said that he and Mr. L. M. Griffiths were the other two living members of the original Committee of the Society. Mr. Dobson recalled the untiring activity and energy of Dr. Shingleton Smith in promoting the interests and welfare of the Society, of which he was the first Secretary, an office he held for fourteen years, during which time he was especially active in promoting pathological and microscopical work at the Society's meetings.

He pointed out how much the medical profession owed to Dr. Smith for the establishment of this successful Society, and in many other ways, especially for constant help and kindness to his junior colleagues, and concluded by wishing him long life in which to enjoy the affection of his many friends.

Mr. Richardson Cross then spoke, and said that his first association with Dr. Shingleton Smith was at King's College, London, when Dr. Smith had just finished a brilliant career as a medical student by gaining the gold medal for the M.B. Lond. Mr. Cross mentioned the part played by Dr. Smith in the foundation of University College, Bristol, the forerunner of the University, and that he was Secretary to the organising Committee of the College. Subsequently he was a most successful teacher in Physiology in the College, first as Lecturer and then as Professor. In 1878 a volume of *Transactions of the Medico-Chirurgical Society* was published, and in 1882 the *Journal* was started with Mr. Greig Smith as Editor and Mr. L. M. Griffiths as Assistant Editor. Mr. Cross mentioned as of particular interest that the first paper in the first volume is a very excellent one by Dr. Shingleton Smith on "Phthisical Contagion," the first of the many able papers for which the *Journal* was subsequently indebted to him, and by which its reputation was enhanced.

In 1892 Mr. Greig Smith resigned the Editorship, and it was felt that Dr. Shingleton Smith by his scientific reputation and his eminent services to the Medico-Chirurgical Society and the *Journal* was marked out for the post. This he undertook with the valuable help of Mr. L. M. Griffiths as Assistant Editor and of a Committee of which the speaker was one of the original members.

He had therefore been Editor for more than twenty years, and under his able guidance the *Journal* had prospered: he leaves it in a stronger position than when he took up his task, and its prosperity is due to the zeal, judgment, and unremitting care which he has devoted to it.

Another benefit which the local profession owed to the foundation of the Medico-Chirurgical Society was the Medical Library, now housed in one of the buildings of the University; this from small beginnings had developed into a fine library, and incidentally owed much to the *Journal*, which has kept it supplied with the large number of medical journals on its exchange list, and has always made over to it the books received for review.

Professor Walter Swayne then on behalf of the subscribers to the testimonial asked Dr. Smith to accept a picture by Reginald Smith, an antique silver salver with a suitable inscription, and an album containing the names of the donors, in token of their recognition of his excellent work as Editor of the *Journal* for so many years.

Dr. Shingleton Smith made a suitable and feeling reply, in which he expressed his appreciation of the gifts, and said that they would always remind him of the cordial and pleasant terms on which he had ever been with the members of the Society and the Committee of the *Journal,* and would be, therefore, an abiding source of gratification to him and to his family.

The rest of the evening was devoted to a smoking concert, in which recitations and songs were given by members of the Society and others, with *inter alia* a most humorous address by Dr. Munro Smith, illustrated by lantern slides, on "Medical Oddities."

Great credit is due to the organisers for the signal success of the evening's entertainment, which they spared no trouble to ensure.

## Figures and Tables

**Figure f1:**